# *N*-[4-(*N,N,N*-Trimethylammonium)Benzyl]Chitosan Chloride as a Gene Carrier: The Influence of Polyplex Composition and Cell Type

**DOI:** 10.3390/ma14092467

**Published:** 2021-05-10

**Authors:** Sergei V. Raik, Tatiana V. Mashel, Albert R. Muslimov, Olga S. Epifanovskaya, Mikhail A. Trofimov, Daria N. Poshina, Kirill V. Lepik, Yury A. Skorik

**Affiliations:** 1 Institute of Macromolecular Compounds of the Russian Academy of Sciences, Bolshoi pr. VO 31, 199004 Saint Petersburg, Russia; raiksv@gmail.com (S.V.R.); poschin@yandex.ru (D.N.P.); 2 Department of Applied Optics, ITMO University, Kronverkskiy pr. 49, 197101 Saint Petersburg, Russia; t.v.mashel@gmail.com; 3 R.M. Gorbacheva Research Institute of Pediatric Oncology, Hematology and Transplantation, Pavlov University, Lva Tolstogo 6/8, 197022 Saint Petersburg, Russia; albert.r.muslimov@gmail.com (A.R.M.); epif-olga@rambler.ru (O.S.E.); lepikkv@gmail.com (K.V.L.); 4 Renewable Energy Laboratory, St. Petersburg Academic University, Khlopina 8/3 lit. A, 194021 Saint Petersburg, Russia; mihail.trofimov@pharminnotech.com; 5 “QR.bio”, Voronezhskaya 5 lit. A, 191119 Saint Petersburg, Russia

**Keywords:** chitosan, polyplex, cell transfection, gene delivery

## Abstract

Polyplex-based gene delivery systems are promising substitutes for viral vectors because of their high versatility and lack of disadvantages commonly encountered with viruses. In this work, we studied the DNA polyplexes with *N*-[4-(*N,N,N*-trimethylammonium)benzyl]chitosan chloride (TMAB-CS) of various compositions in different cell types. Investigations of the interaction of TMAB-CS with DNA by different physical methods revealed that the molecular weight and the degree of substitution do not dramatically influence the hydrodynamic properties of polyplexes. Highly substituted TMAB-CS samples had a high affinity for DNA. The transfection protocol was optimized in HEK293T cells and achieved the highest efficiency of 30–35%. TMAB-CS was dramatically less effective in nonadherent K562 cells (around 1% transfected cells), but it was more effective and less toxic than polyarginine.

## 1. Introduction

Gene therapy is a therapeutic approach that involves the introduction of nucleic acids into a patient’s cells to correct disease. It is an extensively evolving field of medicine that holds significant promise for the treatment of conditions presently considered incurable by conventional approaches, and it is currently being tested in more than 3000 clinical trials worldwide [1,2]. Despite this potential, the introduction of novel gene therapies into clinical practice remains hampered by a lack of efficient and safe gene delivery methods [3,4]. At present, most approved gene therapy products are based on viral delivery. For example, the first FDA-approved gene therapy drug, Luxturna™ [5], was established as a treatment for inherited retinal disease and was based on an adeno-associated viral vector. Zolgensma™ is another that is now indicated for the treatment of spinal muscular atrophy [6].

The prospect of gene therapy has recently been revolutionized by the introduction of ex vivo modified chimeric antigen receptor T (CAR-T) cells for the treatment of resistant B cell malignancies [7]. These cells have also shown promise for solid tumor immunotherapy [8]. Currently, most clinical trials using CAR-T cells use gammaretroviruses and lentiviruses for gene delivery [9]. However, the use of viral vectors has several limitations, including their high production costs [10], regulatory issues [11], and the risk of adverse effects (insertional mutagenesis, immune responses against viral antigens, etc. [12]). In addition, the loading capacity of viral vehicles is also limited [13]. 

For adeno-associated viruses, the most extended length of nucleic acid that can be loaded is about 5 kb, while the limit is around 8–10 kb for lentiviruses and some others. Unfortunately, numerous possible therapeutic constructions, and especially genes, [14] lie far outside that range. For this reason, nonviral gene delivery represents a promising alternative for the delivery of various nucleic acid cargos. However, the low transfection efficiency of nonviral methods remains a bottleneck that hinders their clinical use.

Several ongoing clinical trials are now exploring the delivery of genome editing tools (e.g., TALENs, ZFNs, and CRISPR-Cas9) that can enable a stable correction of the genome with only transient transgene expression [15]. Among the nonviral delivery methods used for these editing tools, electroporation is by far the most popular approach [16,17,18]. However, electroporation is applicable only for ex vivo gene therapy, and its use is limited by possible cell damage and the unspecific transport of cytoplasm and medium components through the membrane [19]. By contrast, lipids and polymer-based gene carriers are among the most versatile delivery tools in terms of structure and physical properties [20,21]. The first polycation investigated for gene delivery was diethylaminoethyl dextran, introduced in the 1960s [22]. In more than 50 years of studies since then, various nature-derived or synthetic polymers with complex architectures have been used for gene delivery [23]. At present, though, only simple polycations such as polyethyleneimine (PEI) have been used in clinical trials [24]. 

One polymer that has been used extensively for gene delivery is chitosan (CS), a cationic polysaccharide characterized by low toxicity, biocompatibility, and high biodegradability [25,26,27]. CS also has amino groups with a pK_a_ of 6.5 that, on the one hand, allow it to serve as a proton sponge that favors transfection by enhancing endosomal escape. On the other hand, the insolubility of CS at pH 7.4 makes it difficult to work with its nonmodified form in most cell culture media [28]. For this reason, chemical modifications were introduced to overcome CS insolubility and to enhance its transfection efficiency [29]. For example, CS derivatives bearing amine-rich substituents, *N*-heterocycles, and alkyl groups have been produced that show enhanced solubility [30,31]. Nevertheless, characterization is a common problem with polymer modifications and, in the case of CS, deacetylation and backbone degradation may occur during the modification steps. Therefore, modification under mild conditions is favored to minimize any effects on the polymer chain. 

In previous studies, we demonstrated the modification of CS by reductive alkylation with aromatic aldehydes [31,32]. These studies showed that the introduction of a trimethylammoniumbenzyl (TMAB) group into CS creates a highly soluble CS polymer with strong DNA affinity. Its transfection efficiency in HEK293T cells increased with increasing polymer excess, as previously demonstrated for PEI [33]. In the present study, we aimed to conduct a more thorough investigation of the effect of the chosen polycation properties, the DNA amount, and the cell type on the final transfection efficiency. The necessity of using a high excess of cationic polymer makes this modification more suitable for ex vivo applications; therefore, a suspension cell line was chosen for transfection to serve as a model of hard-to-transfect but highly clinically relevant cell types, such as T cells or hematopoietic stem cells.

## 2. Materials and Methods

### 2.1. Materials 

The chitosan samples used in this study had the following characteristics: CS37 (Bioprogress, Schelkovo, Russia) had a viscosity average molecular weight (Mη) of 37,000 and a degree of acetylation (DA) of 26%; CS135 (BioLog Heppe Gmbh, Landsberg, Germany) had a Mη of 135,000 and DA of 21%. Branched polyethyleneimine (PEI) had a weight average molecular weight (Mw) of 750,000 and a number average molecular weight (Mn) of 60,000. Polyarginine (PARG, Mw 15,000–70,000) and AlamarBlue™ were purchased from Sigma-Aldrich (St. Louis, MO, USA). The synthesis of 4-formyl-*N,N,N*-trimethylanilinium iodide (FTMA) was as described previously in [32]. Other materials included Dulbecco’s modified Eagle’s medium (DMEM, Lonza, Basel, Switzerland), Roswell Park Memorial Institute medium (RPMI-1640, Lonza, Basel, Switzerland), fetal bovine serum (FBS, HyClone Laboratories Inc, Logan, UT, USA), pmaxGFP Plasmid DNA (pDNA-GFP, Lonza, Basel, Switzerland), and 7-aminoactinomycin D (7AAD, Stemcell Technologies, Vancouver, BC, Canada). Salmon sperm DNA (Derinat™; <1000 bp determined by agarose gel electrophoresis) was purchased from a local pharmacy. 

### 2.2. Synthesis of TMAB-CS

The TMAB-CS samples were synthesized according to a previously published procedure [32]. Briefly, a 2% CS solution in 1% acetic acid was prepared, and its pH was adjusted to 6–7 with 5% NaHCO_3_ solution. A given amount of FTMA (Table 1) was added, and the reaction mixture was stirred at room temperature for 2 h. Subsequently, a threefold molar amount of NaBH_4_ was added. The resulting TMAB-CS was precipitated with acetone, dialyzed for 24 h against 1% NaCl and then again for 72 h against deionized water, and freeze-dried.

### 2.3. NMR Measurements

^1^H NMR spectra of the TMAB-CS samples were recorded on a Bruker 400 MHz UltraShield spectrometer with 9.4 T field. Solutions were prepared in 1% CF_3_COOH in D_2_O. Spectra were recorded at 343 K using a zgpr pulse sequence. ^1^H NMR spectra are provided in the supplementary materials (Supplementary Materials, Figures S1–S4).

### 2.4. Preparation of Polyplexes

The TMAB-CS solution (1 mg/mL) and pmax GFP-DNA solution (0.1 mg/mL) were filtered through sterile syringe filters (0.22 μm) and mixed in the following sequence: TMAB-CS solution was added to phosphate buffered saline (PBS; the volume was calculated to obtain a total volume of 550 μL). The pDNA solution was then added, mixed by vortexing, and left at room temperature.

### 2.5. Cell Culture 

Human embryonic kidney HEK293T cells and the human immortalized myelogenous leukemia K562 cell line were obtained from the Russian Collection of Cell Cultures, Institute of Cytology of the Russian Academy of Sciences (St. Petersburg, Russia) and were used for flow cytometry analysis after transfection with pDNA. The cells were cultured in DMEM (HEK293T) or RPMI (K562) supplemented with FBS (10%) under standard conditions (37 °C, 5% CO_2_, humidified sterile environment).

### 2.6. Plasmid DNA (pDNA-GFP)

The pmaxGFP Plasmid DNA (pDNA-GFP, Lonza, Basel, Switzerland) was propagated in an *Escherichia* coli DH5α culture. Extraction was performed by endotoxin-free plasmid DNA purification using a Plasmid Endofree MaxiPrep Kit (QIAGEN GmbH, Hilden, Germany) from a 400 mL culture (LB with kanamycin 50 µg, 37 °C, 17 h) with 2 preparation cycles. The concentration and quality of the pDNA were evaluated by spectrophotometry using NanoDrop^®^ ND-1000 (Thermo Scientific, Waltham, MA, USA).

### 2.7. Flow Cytometry Analysis

The transfection efficiencies and the images of GFP-positive cells were evaluated using a flow cytometer BD FACSCanto (Becton, Dickinson and Company, Franklin Lakes, NJ, USA). Fluorescent staining of dead cells was performed by adding 7AAD to the cells prior to flow cytometry analysis. Green (green fluorescent protein (GFP)) and red (7AAD) fluorescence channels were recorded.

### 2.8. Gel Retardation Assay 

Polyplexes were prepared with TMAB-CS:pDNA mass ratios of 0.2:1, 2:1, and 20:1. A 10 μL volume of polyplex solution with DNA gel loading dye was loaded onto 1% agarose gel (30 mL) in TAE buffer solution (tris(hydroxymethyl)aminomethane, sodium acetate, sodium ethylenediaminetetraacetate; pH = 8.3) with 10 μg of EtBr. A 100 ng sample of pDNA was used as a reference. Electrophoresis was run for 30 min at 80 V. The agarose gels were documented with a Bio-Rad ChemiDoc gel imaging and analysis system (Bio-Rad, Hercules, CA, USA). An image was taken of the slab gels under ultraviolet irradiation using a standard gel imaging system.

### 2.9. Light Scattering Measurements

The Z-average hydrodynamic diameter and ζ-potential were measured on a Malvern Zetasizer Nano Series instrument (Malvern Panalytical Ltd, Malvern, UK) equipped with a 4 mW He-Ne laser (wavelength 633 nm) and running DTS software. Measurements were carried out at 25 °C at a 173° scattering angle. 

### 2.10. Ethidium Bromide Displacement Assay

A 2.5 µL volume of EtBr solution in water (2.5 × 10^−3^ M) was added to 1.98 mL of PBS (pH 7.4), followed by addition of 16.7 μL of the pDNA stock solution (0.6 mg/mL). The mixture was then titrated with the polycation solution in PBS (1 mg/mL) from mass ratios of 0.1:1 to 10:1. Measurements were recorded using a Shimadzu RF-5301PC spectrofluorometer (Shimadzu, Kyoto, Japan) at λ_ex_ = 560 nm and λ_em_ = 605 nm. 

### 2.11. Optimization of HEK293T Cell Transfection

The HEK293T cells were seeded in 12-well plates (10^5^ cells/well). The influence of different TMAB-CS:pDNA mass ratios on transfection efficiency was assessed by mixing different volumes of TMAB-CS37-54 solutions in PBS (1 mg/mL) with 50 µL of pDNA solution in PBS (0.1 mg/mL) to obtain TMAB-CS:pDNA mass ratios ranging from 0.2:1 to 100:1 (Table 2). The optimal amount of pDNA per 10^6^ cells was determined by mixing different volumes of TMAB-CS solutions in PBS (1 mg/mL) with different volumes of pDNA solution in PBS (0.1 mg/mL) to obtain a TMAB-CS:pDNA mass ratio of 25:1 (Table 3). 

The polyplex solutions were mixed with culture medium to a volume of 1 mL and added to the attached cells. In both experiments, the cells were incubated for 72 h, stained with 7AAD, and analyzed by flow cytometry.

### 2.12. Transfection of the K562/HEK293T Cells 

K562 or HEK293T cells were seeded in 12-well plates (10^5^ cells/well). A 125 μL volume of TMAB-CS solutions in PBS (1 mg/mL) was mixed with 50 μL of pDNA solution in PBS to give a TMAB-CS:DNA mass ratio of 25:1 and a DNA amount of 50 μg/10^6^ cells. PARG and PEI polyplexes were formed at 4:1 mass ratios. The resulting polyplex solutions were added to the cells, incubated for 15 min, and then the culture medium was added to a final volume of 1 mL. The cells were incubated for 72 h, stained with 7AAD, and analyzed by flow cytometry (data are available in the Supplementary Materials, Figures S5 and S6). 

### 2.13. Cytotoxicity Test 

HEK293T cells (5000 cells/well) were seeded in 96-well plates. Polycation solutions in PBS (1 mg/mL) were mixed with pDNA solution in PBS (0.1 mg/mL) to form polyplexes with 25:1 polycation mass excess. Polyplexes were added to cells in different amounts (12.5, 25, and 37.5 µg DNA per 10^6^ cells). The medium with polyplexes was removed after 1 or 24 h, and the cultures were incubated for 96 h, followed by addition of 20 µL 10× AlamarBlue™ solution. After an additional 3 h incubation, cell viability was determined by spectrophotometry.

## 3. Results and Discussion

### 3.1. Synthesis and Characterization of TMAB-CS

The modification of CS amino groups with a TMAB substituent by reductive alkylation is highly efficient at relatively low degrees of substitution (DS). A two-fold amount of FTMA led to a DS higher than 50%. For the synthesis of TMAB-CS37-26, a ratio of FTMA to CS amino groups of 0.7 resulted in a degree of substitution of 26%. Indeed, in the case of alkylation with alkyl halides, a much higher excess of the alkylating agent is generally used. This high excess of alkylating agents usually leads to degrees of substitution much less than 50% [34].

However, when the DS exceeds about half of the CS amino groups, various effects significantly decrease the efficiency of conjugation. These effects include the electrostatic repulsion of the charged quaternary TMAB substituents and the decrease in the steric accessibility of CS amino groups. The fact that steric hindrances occur at high DS is hypothesized by trivial calculations, showing that only 3% of the CS amino groupsare free at a DS of 71%. This is further illustrated by the deformation of the acetamide proton signal on the ^1^H NMR spectrum (Supplementary Materials, Figure S3). In addition to a singlet at 2.08 ppm, an upfield shifted signal appears at 2.07 ppm, caused by a restriction of methyl group spinning. This asymmetry is easily distinguished from that caused by an inhomogeneous magnetic field because the latter causes the same effects for all the signals in the spectrum. A comparison of the spectra of TMAB-CS37-26 and TMAB-CS37-71 reveals that the substituted H-2 signal shifts downfield from 3.23 to 3.32 ppm. We used the acetyl proton as a reference signal because it is not surrounded by other signals and its intensity is not affected by the residual water signal suppression, as happens for the anomeric proton signals at 4.5–5.2 ppm and for the polysaccharide backbone signals at 3.5–4.2 ppm. The procedure used for DS calculation and full signal assignments were as published previously [32]: acetamide protons signals were used as a reference (2.08 ppm, 3×DA H); DS was calculated from integral intensity of aromatic protons at 7.75–8.00 ppm (Supplementary Materials, Figures S1–S4).

### 3.2. Polyplex Formation and Properties

**Figure 1:**
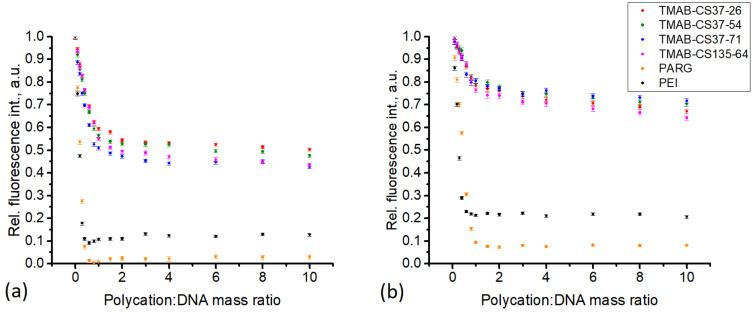
Intercalated EtBr fluorescence quenching: (**a**) pDNA, (**b**) salmon sperm DNA. Data presented as mean ± confidence interval (*p* = 0.05; *n* = 100). Legend is applicable for both graphs.

EtBr fluorescence quenching is a rapid method for determining the affinity of a polycation for DNA [30]. The method of EtBr quenching measurement allows for the determination of a stoichiometric ratio as the intersection of two linear fragments of the curve (a steep fragment describes the specific binding of the polycation to DNA, whereas a shallow fragment refers to nonspecific fluorescence quenching), and it allows for the qualitative assessment of the polycation binding affinity to DNA by the steepness of the curve. Both PARG and TMAB-CS form stoichiometric polyplexes at mass ratios close to 1 (Figure 1). The reason for this is the closeness of the molecular weights of the CS monomeric unit (MW 171.5), the TMAB-substituent (MW 184.5), and the PARG*HCl monomeric unit (MW 192.5). The quenching of EtBr is caused by DNA conformational changes and can therefore correspond to the compaction of the DNA chain. 

**Figure 2:**
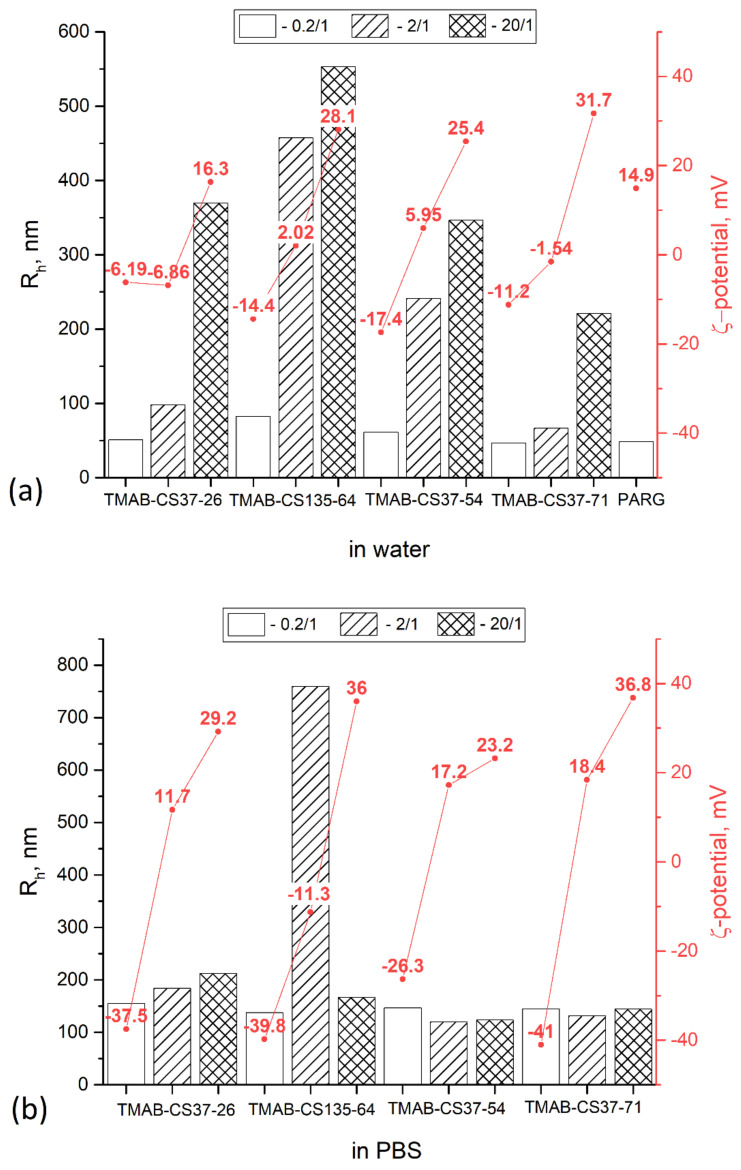
Hydrodynamic radii and ζ-potentials of polyplexes prepared in deionized water (**a**) and PBS (**b**).

The TMAB-CS samples, in all cases, showed similar quenching patterns, with a residual fluorescence of around 65–75% (Figure 1). By contrast, the PARG polyplexes showed a residual fluorescence of around 10%, indicating a higher affinity for DNA. For the pDNA, all the TMAB-CS quenching curves overlapped, and we were unable to see any difference. However, in the case of a short genomic DNA sequence from salmon sperm, we observed both a higher level of fluorescence quenching and a greater curve resolution. The residual fluorescence was 45–55% of the initial level and was correlated with the degree of substitution. Samples with higher DS showed lower residual fluorescence. The same results were observed for PARG (Figure 1). With pDNA, the residual fluorescence level was 10%, whereas with salmon sperm DNA, it decreased to almost zero. The fact that shorter DNA molecules perform differently in this experiment may be explained by the fast compaction of the pDNA. Rapid polyplex formation leaves large fragments of the pDNA chain uncomplexed, so that sites of intercalation remain active. In the case of short DNA chains, their mobility within the polyplex is higher; therefore, more significant conformational changes occur. The high pK_a_ of the guanidine group (13.2) renders it completely charged at physiological pH. These findings are supported by the dynamic light scattering (DLS) data. The PARG:pDNA polyplex (2:1) has a hydrodynamic radius (R_h_) of 50 nm (Figure 2), which is the lowest value among the studied polyplexes at similar ratios. 

DLS is a commonly used method for nanomedicine characterization because it provides rapid results and requires minimal sample preparation. The R_h_ is a hydrodynamic parameter; therefore, it may not correspond to geometric size. Moreover, the measurements at one scattering angle give only the apparent hydrodynamic radius, which is more of a qualitative than a quantitative size parameter. Changes in R_h_, however, can provide useful information about changes in the system. The R_h_ of TMAB-CS:pDNA polyplexes in PBS does not significantly change with the ζ-potential, and both negatively charged and positively charged polyplexes have R_h_ values between 100 and 200 nm. TMAB-CS135-64 at a 2:1 ratio formed large aggregates. In deionized water, the R_h_ of the polyplexes increased with increasing amounts of TMAB-CS, possibly due to the electrostatic repulsion of the polimeric chains. In PBS, this polyelectrolyte effect is suppressed by the shielding of charges provided by the added electrolytes. 

**Figure 3:**
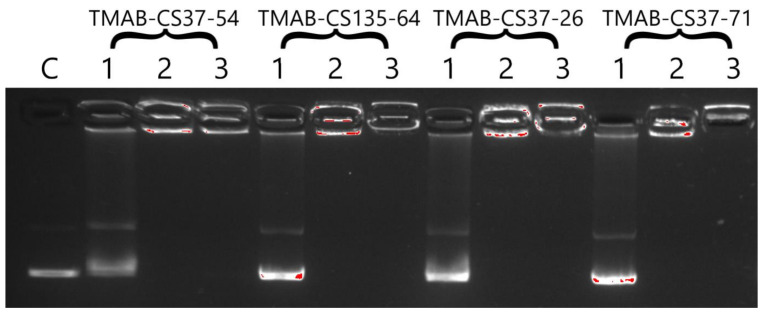
Gel retardation assay for TMAB-CS:DNA polyplexes with different mass ratios (1 = 0.2:1; 2 = 2:1; 3 = 20:1).

Gel retardation assays (Figure 3) showed that the formation of polyplexes with excessive amounts of cationic polymer (2:1 and higher) resulted in no migration of free DNA in the gel.

### 3.3. Optimization of HEK293T Cell Transfection

**Figure 4:**
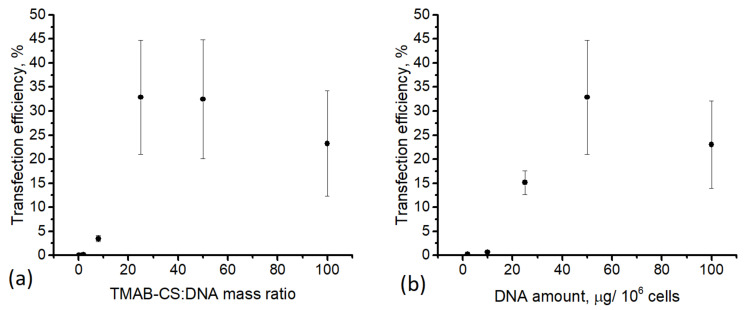
Fraction of GFP expressing HEK293T cells after transfection with TMAB-CS37-54 polyplexes: (**a**) the amount of DNA was 50 μg/10^6^ cells; (**b**) the TMAB-CS:DNA mass ratio was 25:1.

We sought the optimal conditions for comparing different polymers in our cell lines by preparing polyplexes of TMAB-CS37-54 with pDNA and transfecting HEK293T cells with varying amounts of pDNA at different TMAB-CS:pDNA ratios. The number of cells expressing GFP increased with increasing pDNA amounts and reached a maximum at 50 μg per 10^6^ cells (25:1 TMAB-CS:pDNA mass ratio, Figure 4). The TMAB-CS excess increased the transfection efficiency with an optimal ratio of 25:1, indicating its high transfection efficiency and low cytotoxicity. Interestingly, an increase in the ratio from 8:1 to 25:1 caused an increase in cell transfection by an order of magnitude. This nonlinear dependence favors the hypothesis that free cationic chains not only affect endosomal escape by membrane disruption but they also neutralize the cell surface anionic glycans—a step that is also critical for transfection [35].

### 3.4. Transfection of K562 and HEK293T Cells

We examined how the cationic polymer structure affects the transfection efficiency using TMAB-CS samples that differed in DS and chain length, while using PARG and PEI as commercially available controls (Table 4). PEI is considered as a “gold standard” DNA transfection reagent with well-characterized efficiency for both cell lines and primary mammal cells [36,37]. The potential of PARG for genetic material delivery was also described in the literature [38], and it was extensively used by our group for the synthesis of micro- and nanosized carriers [39,40]. The mass ratios of the control polyplexes were chosen based on previously published optimal conditions [38,41].

TMAB-CS135-64 was the most effective transfection agent for HEK293T cells. TMAB-CS samples synthesized from CS37 were significantly less efficient.

While HEK293T cells are the most popular cell model to use in the preliminary screening of transfection efficiency due to their amenability for expression of exogenous nucleic acids delivered by variety of methods with high yields of protein production, these cells were demonstrated to readily uptake nanoparticles via different internalization mechanisms [42]. By contrast, clinically relevant cells for ex vivo gene therapy (e.g., T-cells, hematopoietic stem cells, etc.) are nonadherent cells. These populations are generally considered hard-to-transfect cell types with biological properties that are very different from those of the adherent HEK293T cell line [43,44]. The explanation for these differences is controversial and includes many factors. It cannot be explained by simple physical interactions, as Keller et al. [43] showed that adherent cultivation of TF-1 cells in fibronectin-coated flasks failed to increase transgene expression, whereas cell cultivation on an adherent cell monolayer increased the transfection efficiency by at least an order of magnitude. This means that cell adhesion on its own is not sufficient to enhance transgene expression and that this expression requires regulation at the transcriptional level.

The K562 suspension cell line is used as an in vitro model of leukemia, and it is much more recalcitrant to transfection under conditions optimized for HEK293T cells. In most cases, the transfection efficiency was less than 1%. The TMAB-CS37-71 polyplexes transfected only about 1.5% of cells, with 83% viability, whereas the efficiency of either PARG or PEI polyplexes was 0.1% or less. The K562 represents a model of hard-to-transfect cell type, with several mechanisms impeding the efficiency of transfection mediated by micro- and nanosized carriers, such as lower surface volume, lower expression of contact molecules, and inactive phagocytosis [45,46]. These obtained results are in agreement with previously published work. The transfection efficiency with DNA and lipid-based or modified PEI-based carriers was less than 5% [47,48]. The RNA interference in K562 cells was also substantially lower compared to that in adherent MDA-MB-231 cells [49].

The cell viability determined by 7AAD staining and flow cytometry corresponded well to data obtained by the colorimetric AlamarBlue test (Table 5). All polyplexes except PEI caused less than 10% cytotoxicity. For PEI, the cytotoxicity was 30–50%. Cell viability was also independent of the time of incubation with the polyplexes.

Overall, TMAB-CS performed well as a promising tool for ex vivo gene delivery applications. It showed very moderate cytotoxicity, even at high concentrations, and was able to transfect even the poorly transfectable K562 cell line. Moreover, TMAB-CS provides the possibility to vary the DNA affinity by regulating the DS and therefore the charge density. This allows for the ready modification of gene delivery systems for different cell lines. 

## 4. Conclusions

This study illustrated several important ideas for consideration when designing gene delivery systems. While almost half of the adherent HEK293T cells expressed GFP, the transfection efficiency was more than one order of magnitude lower for the nonadherent K562 cells, underlining the necessity of further optimization of the carrier characteristics for applications that include hard-to-transfect cell types. The transfection efficiency in HEK293T cells increased linearly with increasing amounts of DNA added, but it subsequently reached a plateau at 50 μg DNA/10^6^ cells, and no further increase was observed. The TMAB-CS:DNA mass ratio had the strongest influence on transfection. At ratios lower than 25:1, less than 5% of the HEK293T cells expressed GFP, whereas at ratios of 25:1 and higher, the efficiency was around 30%. TMAB-CS with a higher molecular weight was more effective in HEK293T cells, whereas highly substituted TMAB-CS37-71 was the most effective for K562 transfection.

## Figures and Tables

**Table 1:** Synthesis conditions and degrees of substitution (DS) of *N*-[4-(*N,N,N*-trimethylammonium)benzyl]chitosan chloride (TMAB-CS) samples.

Sample	FTMA:CS Molar Ratio	DS, %
TMAB-CS37-26	0.5	26
TMAB-CS37-54	2	54
TMAB-CS135-64	2	64
TMAB-CS37-71	2 × 2 ^1^	71

^1^ Synthesis was carried out in two steps with a two-fold excess of 4-formyl-*N,N,N*-trimethylanilinium iodide (FTMA). After the first stage, TMAB-CS was precipitated with acetone and redissolved in water, and then the second reaction step was carried out.

**Table 2:** Volumes of TMAB-CS37-54 solutions for different TMAB-CS:pDNA mass ratios.

**TMAB-CS:pDNA Mass Ratio**	100:1	50:1	25:1	10:1	2:1	0.2:1
**TMAB-CS, µL**	500	250	125	50	10	1
**pDNA, µL**	50	50	50	50	50	50

**Table 3:** Volumes of TMAB-CS37-54 and pDNA solutions for a 25:1 mass ratio.

**pDNA Amount, µg/10^6^ Cells**	100	50	25	10	2	0.2
**TMAB-CS37-54, µL**	250	125	62.5	25	5	1
**pDNA, µL**	100	50	25	10	2	0.2

**Table 4:** Transfection and cytotoxicity of polyplexes to HEK293T and K562 cells.

No.	Polyplex	HEK293T		K562	
		Transfection Efficiency, % ^1^	Toxicity, %	Transfection Efficiency, % ^1^	Toxicity, %
**1**	TMAB-CS37-54	18	4.3	0.1	5.0
2	TMAB-CS135-64	50	3.7	0.5	15
3	TMAB-CS37-26	2	2.5	0	6.2
4	TMAB-CS37-71	21	2.7	1.5	17
5	PEI (4:1)	16	48	0.1	8.0
6	PARG (4:1)	11	2.4	0	19
7	Control	0	1.5	0	3.3

^1^ Transfection efficiency is the fraction of viable cells expressing green fluorescent protein (GFP). Flow cytometry data are available in the Supplementary Materials, Figures S5–S6.

**Table 5:** Cell viability (% ± SD; *n* = 3) determined by the AlamarBlue™ assay.

Time	1 h			24 h		
DNA amount, µg/10^6^ cells	**12.5**	**25.0**	**37.5**	**12.5**	**25.0**	**37.5**
TMAB-CS37-71	93 ± 4	89 ± 1	93 ± 4	97 ± 1	94 ± 1	98 ± 3
TMAB-CS37-26	96 ± 6	94 ± 1	94 ± 1	95 ± 1	95 ± 1	94 ± 3
TMAB-CS135-64	97 ± 6	96 ± 2	98 ± 3	99 ± 0.2	99 ± 1	102 ± 1
TMAB-CS37-54	108 ± 4	104 ± 2	103 ± 3	102 ± 0.1	100 ± 0.1	98 ± 0 6
PARG	91 ± 4	89 ± 1	89 ± 2	96 ± 1	92 ± 1	94 ± 3
PEI	70 ± 2	61 ± 3	63 ± 2	59 ± 1	53 ± 1	55 ± 2

## Data Availability

Data are contained within the article and Supplementary Materials.

## Supplementary Materials

The following are available online at https://www.mdpi.com/article/10.3390/ma14092467/s1, Figure S1: ^1^H NMR spectrum of TMAB-CS37-26 (400 MHz, 343 K), Figure S2: ^1^H NMR spectrum of TMAB-CS37-54 (400 MHz, 343 K), Figure S3: ^1^H NMR spectrum of TMAB-CS37-71 (400 MHz, 343 K), Figure S4: ^1^H NMR spectrum of TMAB-CS135-64 (400 MHz, 343 K), Figure S5: Flow cytometry data for HEK293T transfection (No. 1–6 from Table 4 in the same order), Figure S6: Flow cytometry data for K562 transfection (No. 1–6 from Table 4 in the same order).
