# Supplementary material for: N-[4-(N,N,N-Trimethylammonium)Benzyl]Chitosan Chloride as a Gene Carrier: The Influence of Polyplex Composition and Cell Type
Source: Materials (Basel). 2021 May 10;14(9):2467. doi: 10.3390/ma14092467 (PMC8126137; doi:10.3390/ma14092467)
Supplement: Supplementary file 1 [file materials-14-02467-s001.zip › materials-1190575-supplementary.pdf]

materials

Article

N-[4-(*N*,*N*,*N*-Trimethylammonium)Benzyl]Chitosan Chloride as a Gene Carrier: The Influence of Polyplex Composition and Cell Type

Sergei V. Raik [1], Tatiana V. Mashel [2], Albert R. Muslimov [3,4,5], Olga S. Epifanovskaya [3], Mikhail A. Trofimov [4], Daria N. Poshina [1], Kirill V. Lepik [3] and Yury A. Skorik [1,*]

1 Institute of Macromolecular Compounds of the Russian Academy of Sciences, Bolshoi pr. VO 31, 199004 Saint Petersburg, Russia; raiksv@gmail.com (S.V.R.); poschin@yandex.ru (D.N.P.)
2 Department of Applied Optics, ITMO University, Kronverkskiy pr. 49, 197101 Saint Petersburg, Russia; t.v.mashel@gmail.com
3 R.M. Gorbacheva Research Institute of Pediatric Oncology, Hematology and Transplantation, Pavlov University, Lva Tolstogo 6/8, 197022 Saint Petersburg, Russia; albert.r.muslimov@gmail.com (A.R.M.); epif-olga@rambler.ru (O.A.E.); lepikkv@gmail.com (K.V.L.)
4 Renewable Energy Laboratory, St. Petersburg Academic University, Khlopina 8/3 lit. A, 194021 Saint Petersburg, Russia; mihail.trofimov@pharminnotech.com
5 "QR.bio", Voronezhskaya 5 lit. A, 191119 Saint Petersburg, Russia; albert.r.muslimov@gmail.com
* Correspondence: yury_skorik@mail.ru

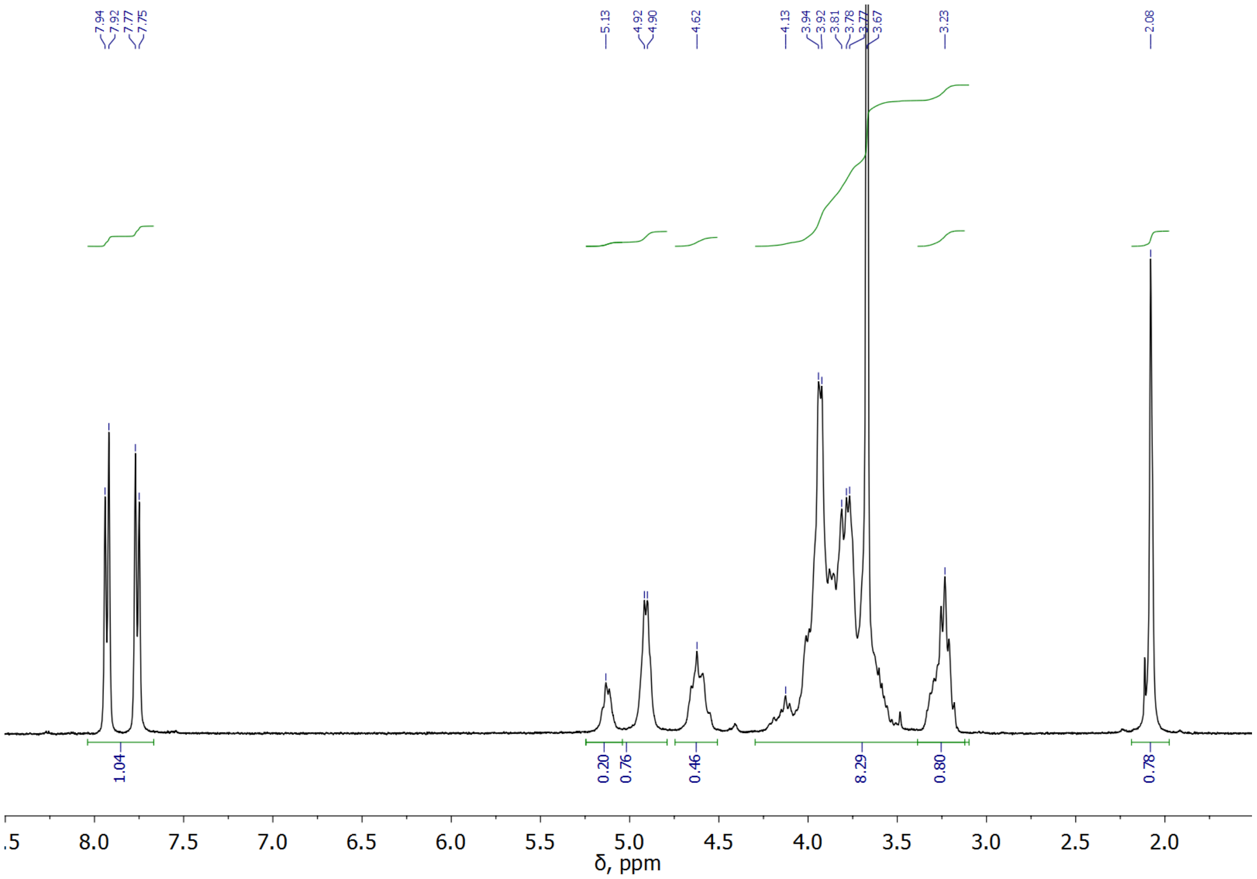

Figure S1. ^{1}H NMR spectrum of TMAB-CS37-26 (400 MHz, 343 K).

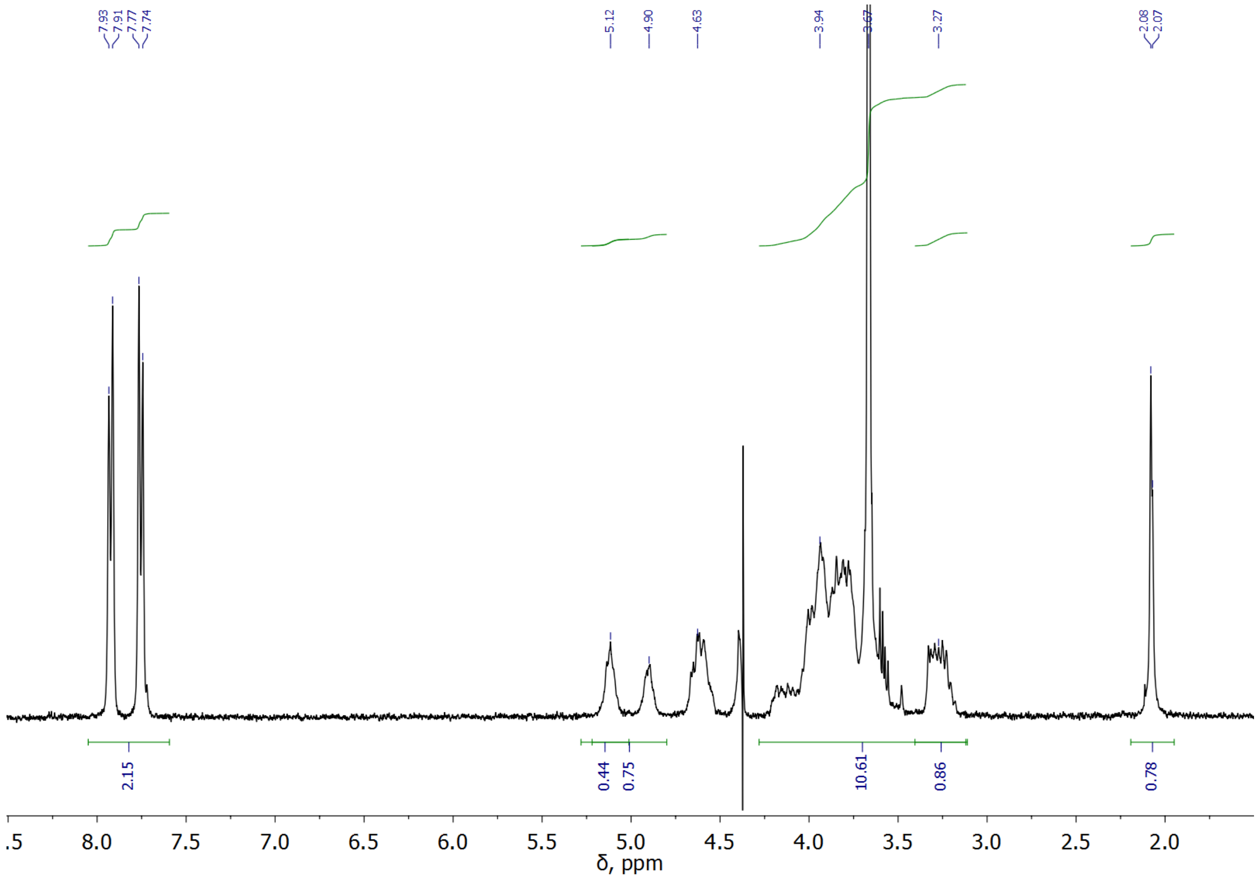

Figure S2. ^{1}H NMR spectrum of TMAB-CS37-54 (400 MHz, 343 K).

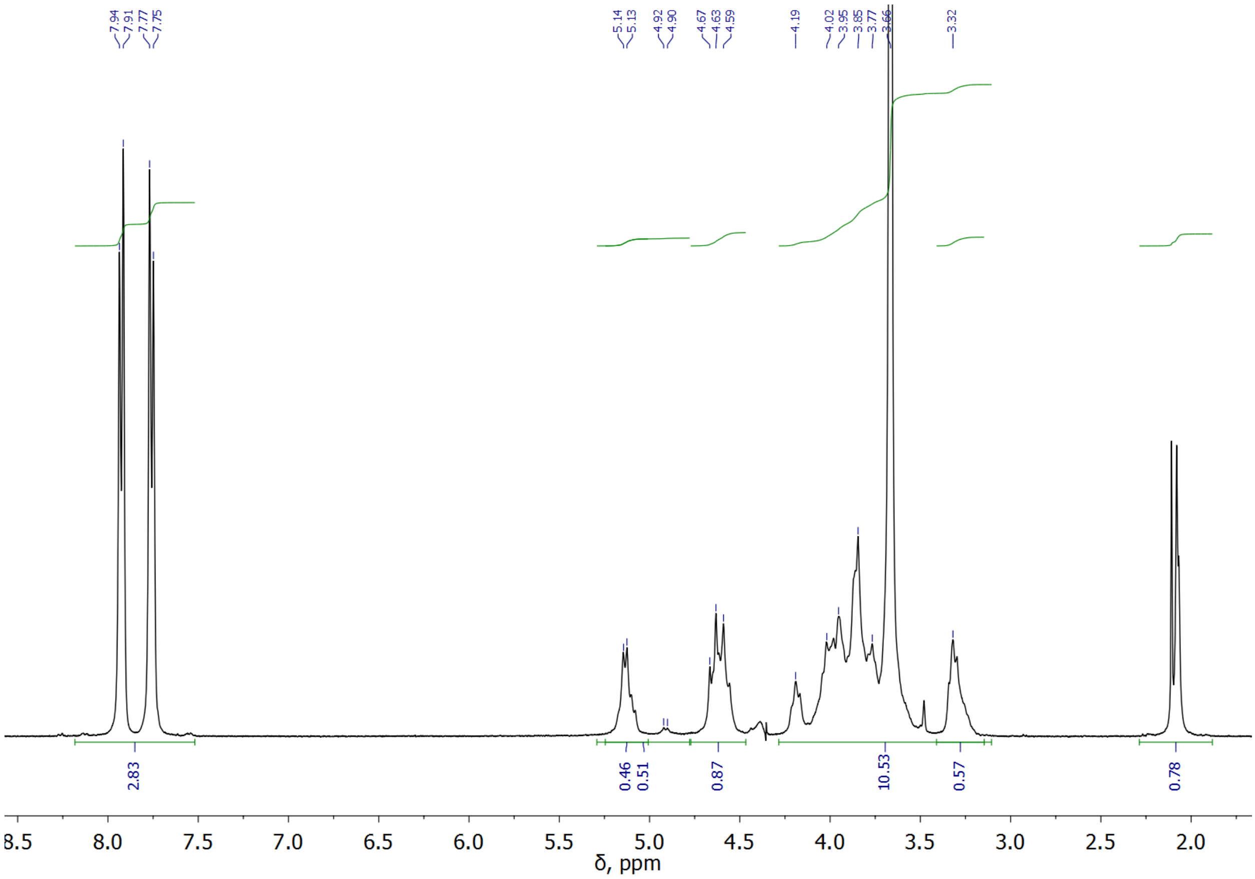

Figure S3. ^{1}H NMR spectrum of TMAB-CS37-71 (400 MHz, 343 K).

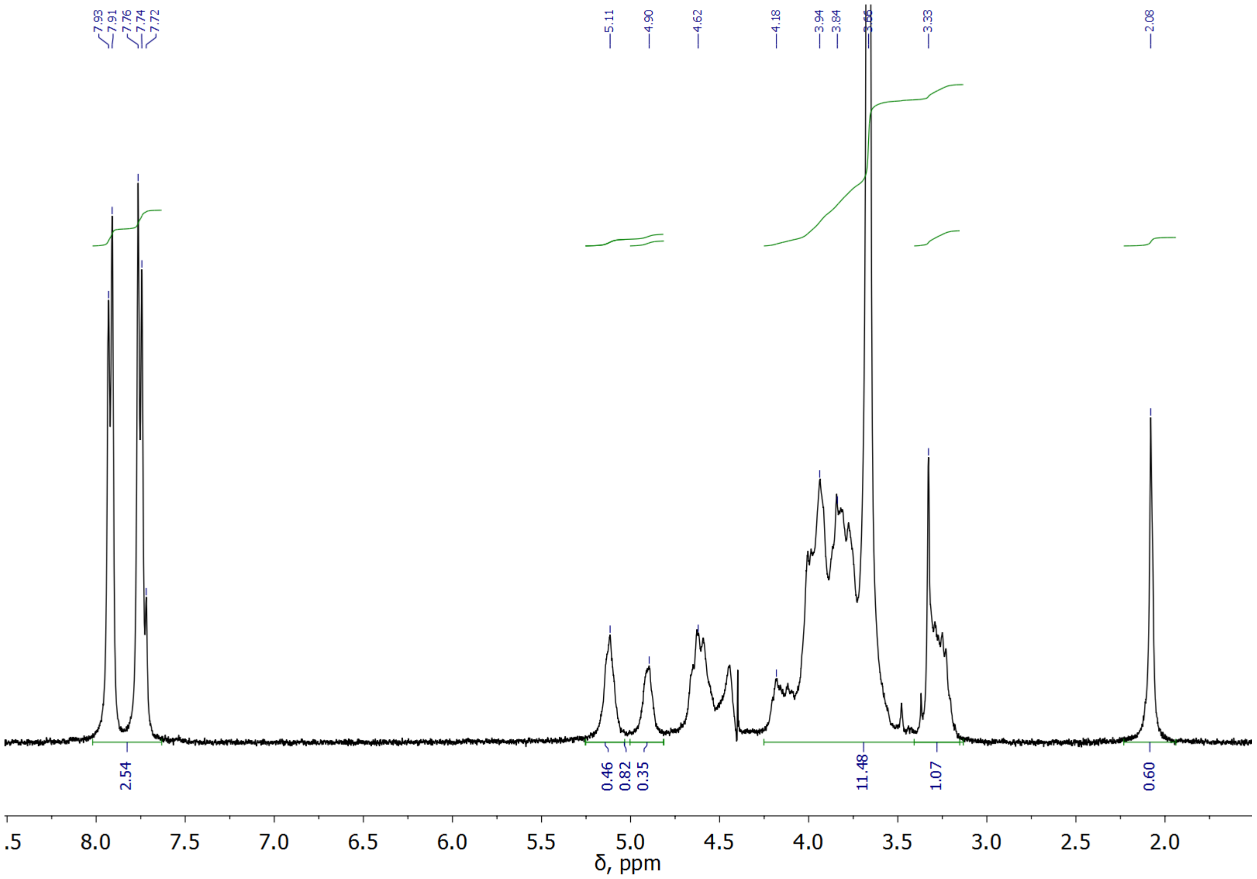

Figure S4. ^1H NMR spectrum of TMAB-CS135-64 (400 MHz, 343 K).

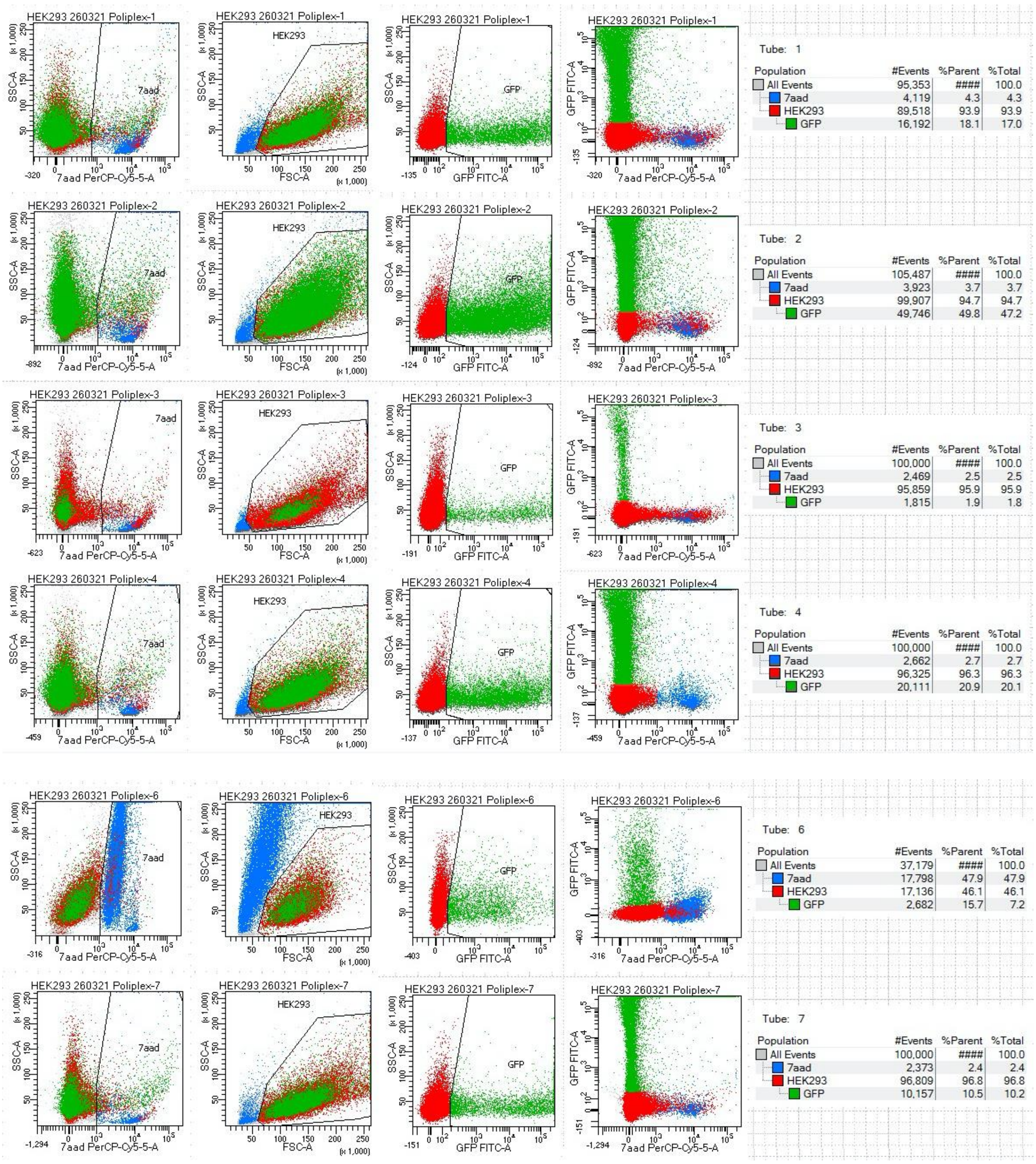

Figure S5. Flow cytometry data for HEK 293T transfection (No. 1-6 from Table 4 in the same order).

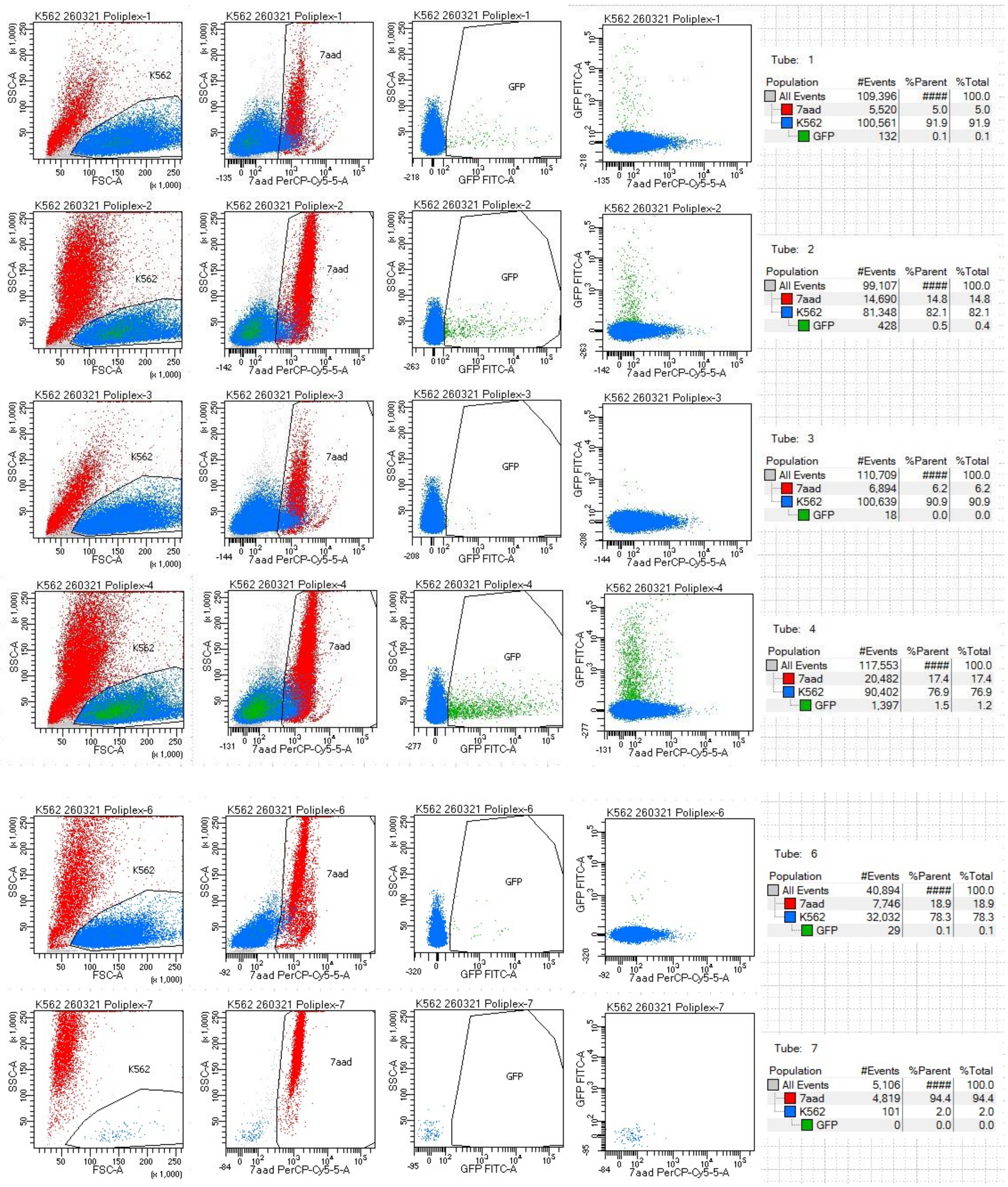

Figure S6. Flow cytometry data for K562 transfection (No. 1-6 from Table 4 in the same order).
